# Vegetation Greenness Dynamics in the Western Greater Khingan Range of Northeast China Based on Dendrochronology

**DOI:** 10.3390/biology11050679

**Published:** 2022-04-28

**Authors:** Jibin Dong, Tingting Yin, Hongxiang Liu, Lu Sun, Siqi Qin, Yang Zhang, Xiao Liu, Peixian Fan, Hui Wang, Peiming Zheng, Renqing Wang

**Affiliations:** 1 Institute of Ecology and Biodiversity, School of Life Sciences, Shandong University, Qingdao 266237, China; 201912331@mail.sdu.edu.cn (J.D.); 202012553@mail.sdu.edu.cn (T.Y.); 202112485@mail.sdu.edu.cn (H.L.); 201932351@mail.sdu.edu.cn (L.S.); 201932349@mail.sdu.edu.cn (S.Q.); 202012584@mail.sdu.edu.cn (Y.Z.); flippedlx@sdu.edu.cn (X.L.); wanghui1227@sdu.edu.cn (H.W.); rqwang@sdu.edu.cn (R.W.); 2 Shandong Provincial Engineering and Technology Research Center for Vegetation Ecology, Shandong University, Qingdao 266237, China; 3 Qingdao Forest Ecology Research Station of National Forestry and Grassland Administration, Qingdao 266237, China; qdlinye@163.com

**Keywords:** dendrochronology, the Greater Khingan Range, vegetation greenness dynamics, climate change, human activities

## Abstract

**Simple Summary:**

This study reconstructed 193 years of vegetation greenness dynamics in the study area based on the chronology of the tree rings of *Pinus sylvestris* var. *mongolica* Litv. in the western Greater Khingan Range, and analyzed the vegetation greenness dynamics pattern accordingly. After the 1950s, it was found that the scale and intensity of extreme changes in the vegetation greenness dynamics increased significantly, and the frequency of occurrence also increased significantly. The study also found that climate (precipitation) is the most important factor influencing the change of vegetation greenness dynamics in the western Greater Khingan Range; the influence of human activities is also important and rapid, and the degree of influence has gradually increased in recent years. Finally, the influence of human activities on the vegetation greenness dynamics was ranked in terms of importance, as follows: livestock > afforestation > population > farmland.

**Abstract:**

Understanding the vegetation greenness dynamics in the forest–steppe transition zone is essential for ecosystem management, and in order to study ecological changes in the region. This study provides a valuable record of the vegetation greenness dynamics in the western Greater Khingan Range over the past 193 years (1826–2018) based on tree-ring data represented by the normalized difference vegetation index (NDVI). The reconstructed vegetation greenness dynamics record contains a total of 32 years of high vegetation greenness and 37 years of low vegetation greenness, together occupying 35.8% of the entire reconstructed period (193 years). Climate (precipitation) is the main influence on the vegetation greenness dynamics at this site, but human activities have also had a significant impact over the last few decades. The magnitude, frequency, and duration of extreme changes in vegetation greenness dynamics have increased significantly, with progressively shorter intervals. Analyses targeting human behavior have shown that the density of livestock, agricultural land area, and total population have gradually increased, encroaching on forests and grasslands and reducing the inter-annual variability. After 2002, the government implemented projects to return farmland to its original ecosystems, and for the implementation of new land management practices (which are more ecologically related); as such, the vegetation conditions began to improve. These findings will help us to understand the relationship between climate change and inter- and intra- annual dynamics in northeastern China, and to better understand the impact of human activities on vegetation greenness dynamics.

## 1. Introduction

Changes in precipitation patterns due to global warming have increased the frequency and intensity of extreme weather events [1]. The prediction of future climate change in the sixth report of IPCC indicates that the global temperature will rise further and the frequency of extreme events will also increase [2,3]. The increase of extreme events will cause damage to vegetation, especially the vegetation in the transition zone between forest and grassland, affecting vegetation coverage [4,5,6,7]. Vegetation is the main constituent element of terrestrial ecosystems, and it plays an important role in global ecological functions [8]. Therefore, it is of great significance to study the driving force of the dynamic changes of vegetation greenness in order to analyze the ecological changes in the context of global warming [9].

Tree rings have the advantages of accurate dating, strong continuity, and sensitivity to the environment, and have become one of the most important methods to study natural geographical changes [10,11]. A large number of studies use tree-ring width as a surrogate to study the dynamics of vegetation greenness, in order to fill the gap of normalized vegetation index (NDVI) data and obtain the dynamic change law of historical vegetation greenness [12,13]. The climate and environmental factors in the same area jointly affect the vegetation [14], such that the change of tree-ring width may have a certain correlation with the change of vegetation greenness. In the existing studies, few have analyzed the impact of human factors on the dynamics of vegetation greenness, such that the relationship between human activities and vegetation is still unclear [15,16,17]. Therefore, studying the dynamic characteristics of vegetation greenness in long time series and their relationship with climate change and human activities will not only help to understand the mechanism of vegetation greenness change, but also provide reference data for vegetation greenness models. This will help us to better predict future changes in vegetation greenness and deal with the impact of future extreme events on the dynamics of vegetation greenness [10,18].

Located in the northeastern region of China, Hulunbuir features a higher latitude and experiences greater warming than other regions. The warming of the region can exacerbate ecological and environmental problems in the terrestrial ecosystem of the area [19,20]. The short history of existing observations of vegetation greenness dynamics in this area limits a comprehensive understanding of the characteristics and driving forces. Consequently, it is important to study the relationship between vegetation greenness dynamics and climate change in this region.

This study is based on the hypothesis that the NDVI (as a proxy of LAI) and tree-ring width are similarly affected by climatic conditions, and are thus expected to be correlated. Based on the above background, the following research objectives were derived: (1) to investigate the correlation between tree-ring width and NDVI, and to reconstruct the past vegetation greenness dynamics using tree-ring data; (2) to investigate the change pattern of vegetation greenness dynamics, and the main distribution periods of extreme change periods; (3) to investigate the influence mechanism of climate and human activities on vegetation greenness dynamics.

## 2. Materials and Methods

### 2.1. Study Region

**Figure 1:**
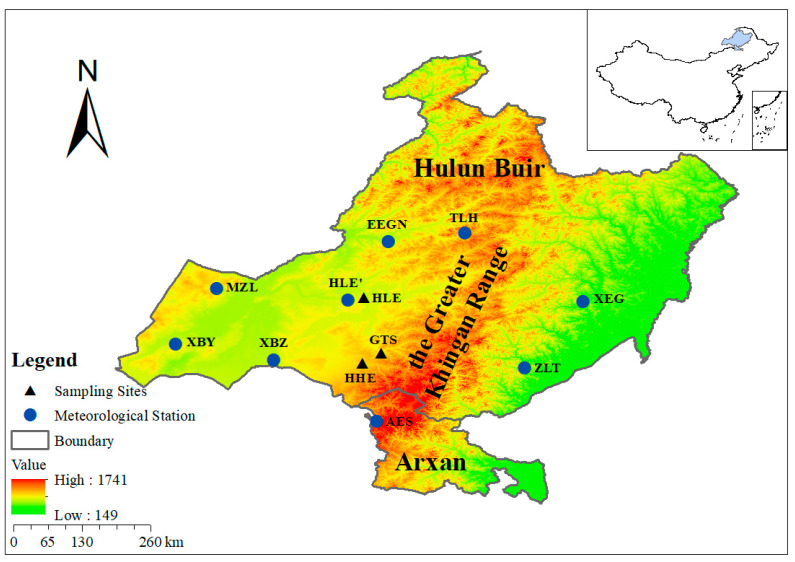
Location of the sampling sites and meteorological stations (sampling site: HLE, Haila’er; GTS, Guangtoushan; HHF, Huihe Forestry; meteorological station, XBY, Xinbalhoo Youqi; XBZ, Xinbalhoo Zuoqi; MZL, Manzhouli; EEGN, Eerguna; TLH, Tulihe; XEG, Xiaoergou; ZLT, Zhalantun; AES, Aershan).

The study area was located in Hulunbuir City and Arxan City in northeastern Inner Mongolia (115°13′–126°04′ E, 47°05′–53°20′ N; Figure 1), situated in the forest–grass transition zone. The site is located at the boundary line between the second and the third orders in China, at the boundary line between semi-humid and semi-arid monsoon and non-monsoon climates, and at the 400 mm equivalent precipitation line. It is an extremely important geographical boundary line in China, with a sensitive climate that makes it suitable for tree-ring analysis.

**Figure 2:**
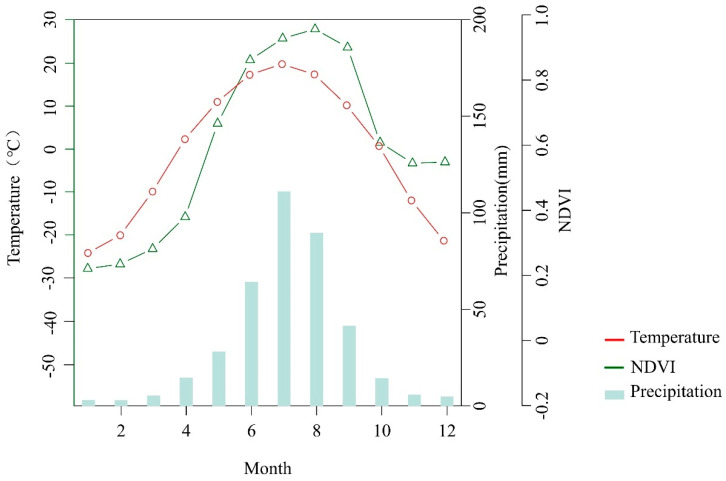
The monthly mean temperature (circle curve), monthly mean NDVI (triangle curve), and monthly total precipitation (bar graph) for the study area were obtained from the available records of nine meteorological stations; time span: 1951−2019.

This area has a temperate continental climate. Due to the topography of the Great Khingan Range and its distance from the sea, humid air masses reach the area infrequently; as a result, the region is dry, with little rain, while at the same time being hot. Precipitation in the study area is mainly concentrated in the summer, with a mean annual temperature of −0.7 °C and a mean annual precipitation of 386.4 mm (Figure 2). The period of the year with the highest NDVI values is from May to September, and the period with the lowest vegetation greenness dynamics is concentrated in the winter months. This study took place in the western Greater Khingan Range, in the transition zone with the Hulunbuir grassland. In this area there is a large amount of agricultural and construction land (mainly from the reclamation of forest and grassland); livestock rearing is more primitive, and is carried out on a large scale.

### 2.2. Dataset

(1) The tree-ring chronology

All of the tree cores were collected using *Pinus sylvestris* var. *mongolica* Litv. The tree-ring samples were collected from Haila’er National Forest Park, Honghuaerji Zhangzisong National Forest Park, and Huihe Forestry (Figure 1, Table 1). The tree cores were drilled using increment borers according to standard procedures defined by the International Tree Dendrochronology Database (ITRDB) [21,22]. The tree-ring width was measured using LINTAB 6 (0.001 mm); COFECHA was cross-checked for dating [23], and then the site-level chronology was developed using ARSTAN [21,24]. Negative exponential or linear functions were used to remove the tree growth trends. The statistical features of the standard chronology (STD) are shown in Table 2.

**Figure 3:**
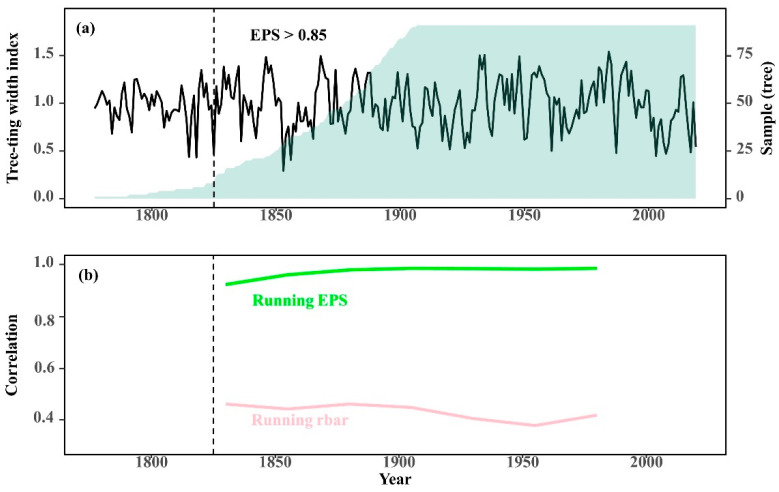
Regional tree-ring standard chronology (HLBE_std_) and sample size (**a**); run Rbar (run based on a 50-year window with a lag of 25 years), run Expressed Population Signal (EPS) (**b**); EPS > 0.85 determines the chronology reliability period.

A regional chronology (HLBE_std_) was created using a total of 159 core samples from the three sampling locations after excluding shorter or decayed cores (Table 1). The regional chronology spans the years 1777 to 2019, with a reliable interval of 1826–2019 for EPS > 0.85 (Figure 3).

(2) Climate datasets

Meteorological data for the study area were obtained from the China Meteorological Data Service Centre (http://data.cma.cn/, accessed on 21 June 2021), and included the monthly precipitation and monthly mean temperature for the period 1952–2019. Meteorological records from nine weather stations were averaged to reduce small-scale noise or random components, and to be representative of a wider range of regional climate conditions; we used the arithmetic mean dataset of the nine meteorological stations for further analysis.

(3) NDVI datasets

The NDVI is one of the most frequently used vegetation indices, which can reflect the greenness dynamics, plant phenology and biomass of regional vegetation [25,26]. This study adopts the global GIMMS NDVI3g v1 dataset (1981–2015) (http://data.tpdc.ac.cn/en/data, accessed on 21 June 2021). This NDVI dataset has been corrected for calibration, view geometry, volcanic aerosols, and other effects which are not related to vegetation changes. The temporal resolution of the product is twice per month, and the spatial resolution is 1/12 of a degree. The temporal cover is from July 1981 to December 2015. The resulting NDVI was used to represent regional changes in vegetation greenness dynamics.

(4) Socio-economic datasets

The data types were the total livestock, farmland area, population, and planted forest area. The data were from the Hulunbuir and Arxan City *Government Statistical Yearbook*. The selected human activities include the quantity of livestock, area of afforestation, population, and area of cultivated land. Based on a random forest regression model, the importance of human activities could be assessed from these activities [27]. After ranking human activities in order of importance, the top-ranked fraction of human activities was selected, and these important human activities were some of the factors that were obviously closely associated with vegetation greenness dynamics. The dataset was collected from CNKI (https://cnki.net/, accessed on 21 June 2021).

### 2.3. Statistical Analysis

(1) Reconstruction and examination of the NDVI

The NDVI of the study area was calculated using the maximum synthesis method [28]. The maximum value synthesis method is widely used in data synthesis because it can eliminate the effects of residual clouds, atmospheric scattering and the solar altitude angle from the data. The maximum synthesis method is implemented using the equation b1 × (b1 ge b2) + b2 × (b2 gt b1) with the band operation module of the software ENVI 4.7, where b1 and b2 represent the NDVI data before and after 15 days of each month, respectively, in order to obtain the monthly NDVI dataset. The relationships between the regional chronology, NDVI, and climate data were analyzed using Pearson correlation. NDVI reconstructions were performed using multiple linear regression models [29]. Separate calibration–validation methods were used to verify the stability of the NDVI reconstruction equation using error-reduction values (RE), effective coefficients (CE), sign tests (ST), and first-order difference sign tests (ST_1_) [21].

(2) Extreme events analysis

In this study, the definition method and analysis method of extreme events in the field of climate reconstruction were introduced in order to analyze the extreme changes of vegetation greenness in the study site. The detailed methods are as follows:

High NDVI values = NDVI_mean_ + 1 SD (1)

Low NDVI values = NDVI_mean_ − 1 SD (2)

(3) Analysis of the influencing factors

Residual analysis methods were used to calculate the effects of climate and human activities on the vegetation greenness dynamics [30].

The formulae are as follows:

NDVI_total_ = a × Pre + b × Tem + c (3)

NDVI_human_ = NDVI_total_ − NDVI_climate_ (4)

Here, NDVI_climate_ refers to the NDVI under the influence of climate obtained from the regression equation reconstructed based on climate records (temperature and precipitation), and NDVI_total_ refers to the regional total NDVI reconstructed using the tree-ring width; a, b, and c are model parameters; and Tem (°C) and Pre (mm) refer to the average temperature and accumulated precipitation in the growing season, respectively. 

The NDVI_human_ is obtained by Formulae (3) and (4), and the contribution rate of human activities is the ratio of the NDVI affected by human activities to the total NDVI. The formula is as follows:

(5) Contributionhuman(%)=NDVIhumanNDVItotal×100%

The importance ranking of the effects of human activities on the vegetation greenness dynamics change was calculated using random forests [27].

## 3. Results and Discussion

### 3.1. Reconstruction and Examination of the NDVI

**Figure 4:**
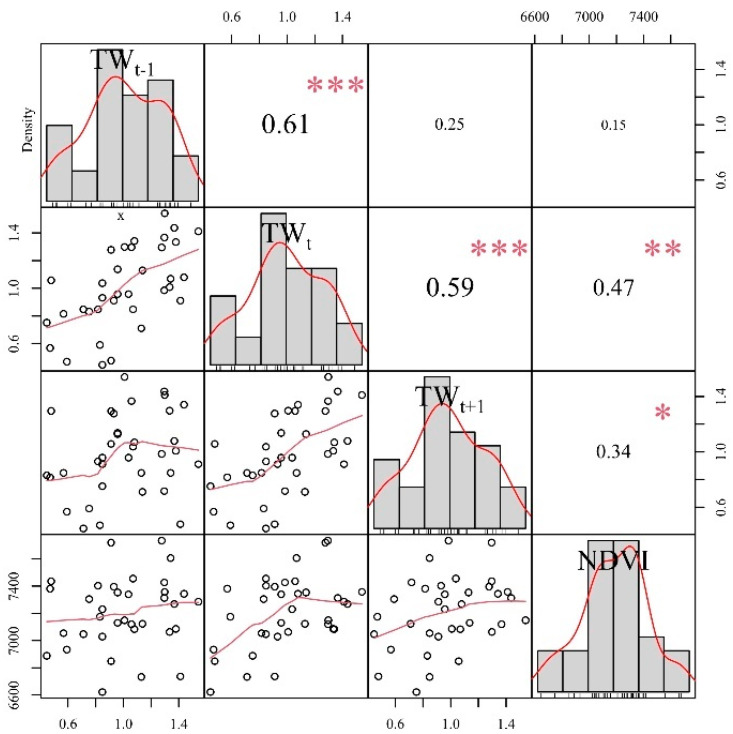
Correlation between the tree-ring index and NDVI. TW_t_ refers to the tree-ring width index of the current year, TW_t−1_ refers to the tree-ring width index of the previous year, TW_t+1_ refers to the tree-ring width index of the next year, the scatter plot refers to the plot of the original data distribution of the tree-ring width index, the bar graph refers to the plot of the frequency distribution of the tree-ring width index, the red line represents its trend, “*”refers to *p* < 0.05, “**”refers to *p* < 0.01, and “***”refers to *p* < 0.001.

Because trees and vegetation in the same area are influenced by factors such as the common climate, changes in the NDVI are correlated with the radial growth of trees. Meanwhile, NDVI can affect the net primary productivity of the forest, and thus the radial growth of trees. As such, tree-ring width is used as an indicator to study changes in NDVI. The correlation results based on tree rings and NDVI_5–9_ indicated that tree rings could represent the vegetation greenness dynamics of the growing season in the western Greater Khingan Range, with correlations as high as 0.47 (Figure 4, *p* < 0.01) and 0.34 (Figure 4, *p* < 0.05) between the tree-ring index and NDVI in the current year and the following year. High NDVI values are closely related to the radial growth of tree rings in the current year and the following year. The tree growth and vegetation greenness dynamics were consistent, suggesting that both were controlled by common climatic factors. In this case, the link is indicative of a relationship between two separate physiological processes occurring within the tree; first, the photosynthetic activity, indirectly measured by the greenness in the canopy (NDVI), and second the carbon allocation (tree-ring width). Furthermore, this implies that there is a common limiting factor, and also that there is a time lag between the processes [31].

The NDVI can affect the next year’s growth of trees. The NDVI in the current year can affect the photosynthesis of trees, which in turn affects the accumulation of organic matter [32], resulting in significant changes in the radial growth of trees. At the beginning of the growing season, the non-structural carbohydrate (NSC) content of coarse tree roots decreased, and the root NSC was provided for tree growth and development. At the end of the growing season, the coarse tree root NSC content increases and NSC is transported to the roots to be stored for the next year’s growth. The coarse roots are the main storage site for non-structural carbohydrates in trees, and are the main source of energy for plant survival in winter and growth in early spring. The NSC accumulated in the roots plays an important role in carbon allocation for tree growth, and to some extent constrains the growth metabolism of above-ground organs [33].

**Figure 5:**
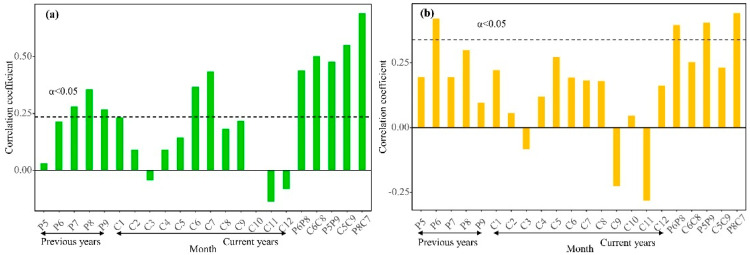
Correlation analysis between the Ring Width Index (RWI), NDVI, and precipitation. Correlation between the RWI and precipitation (**a**); correlation between the NDVI and precipitation (**b**). The horizontal dashed line is the 95% confidence line; Previous year refers to the correlation of precipitation in the previous year, and the month is represented by P; Current years is the correlation of the precipitation in the current year, and the month is represented by C; the alphanumeric combination in the month combination letter represents the year, and the number represents the month.

In order to demonstrate that tree rings could represent the changing vegetation greenness dynamics in the study area, we further analyzed their control by common climatic factors (Figure 5a,b). The results showed that both tree rings and the NDVI presented positive correlations with precipitation during the previous and current growing seasons. This suggests that they were controlled by a common climatic factor (precipitation). The relationship between the temperature and NDVI showed a positive correlation, but it was not significant. Because the study area belongs to a temperate continental climate zone, the temperature has a stronger limiting effect on vegetation compared to moisture. At the same time, the study area has a higher latitude and lower average annual temperature; too low a temperature will limit the growth of plants, such that the increase of temperature can promote the growth of plants to some extent.

The width of the tree-ring represents the growth of the trees, and their growth rate is determined by the net photosynthetic rate. When unfavorable meteorological factors and others such as age, climate, and stand competition, etc., act on vegetation, this will inhibit photosynthesis in plant leaves and slow the radial growth of plants, thereby controlling the tree-ring width and NDVI [34]. Additionally, trees are an important vegetation expression, and their growth state represents vegetation greenness dynamics. Consequently, tree-ring chronology can reflect the dynamics of vegetation greenness during the growing season. Thus, tree-ring width has been used as an indicator to study changes in vegetation greenness. Because the NDVI basically reaches its maximum during the growing season (May–September), we established a transfer function for NDVI from May to September, as follows:

$${\mathrm{NDVI}}_{5-9}=0.041\times {\mathrm{TW}}_{t}+0.026\times {\mathrm{TW}}_{t+1}+0.668,$$

(6) $$(n=34,\;r=0.55,\;R^{2}=0.31,\;R_{\mathrm{adj}}^{2}=0.26,\;p\;<\;0.01),$$

where TW*_t_* is the tree-ring index for a given year, and TW*_t_*_+1_ is the tree-ring index for the previous year. The model explained 31% of the variance in the NDVI data (26% after adjustment for the loss of degrees of freedom) for the period from 1985 to 2015. $R_{\mathrm{adj}}^{2}$ refers to the amount of variance explained after adjusting for degrees of freedom. This removes the effect of the number of independent variables on *R*^2^, such that the magnitude of *R*^2^ only responds to the goodness of fit of the regression equation. The *F* value, *r*, ST, and ST_1_ were all statistically significant, and RE and CE were both positive (Table 3), indicating that the regression model has been statistically validated [21,22].

### 3.2. Extreme Events of Vegetation Greenness Dynamics

**Figure 6:**
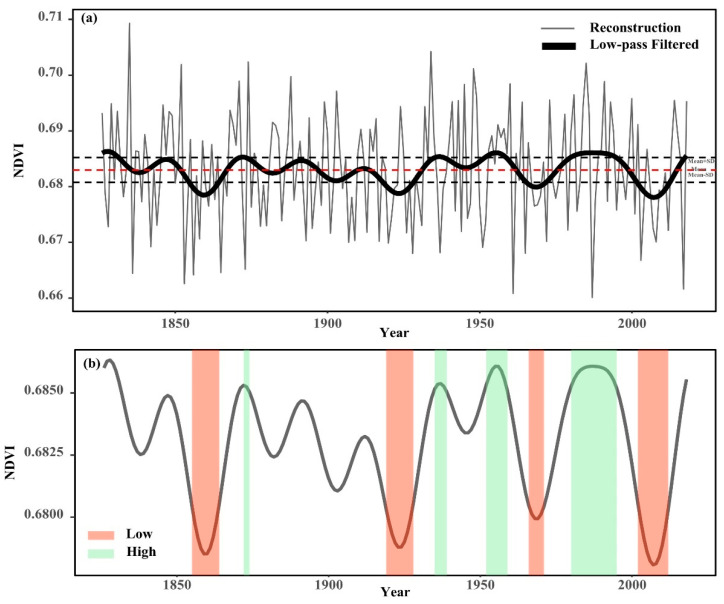
(**a**) NDVI_5–9_ curve after 20 years of low-pass filtering (thick line). The thin line represents the reconstructed NDVI_5–9_, and the horizontal line indicates the mean and mean ± one standard deviation. (**b**) Time interval between the occurrence of extreme events such as low and high cover during the reconstruction interval. The thick lines represent 20 years of low flux data. The red shaded areas represent low cover intervals, and green shaded areas represent high cover intervals.

The vegetation greenness dynamics series illustrates strong decadal-scale changes reflecting a continuous alternation of high and low values. As a result, we used a 20-year low-pass filter to highlight changes over decades. Based on the transfer function and the tree-ring length, we reconstructed the NDVI changes from 1826 to 2018 (Figure 6a). Over the past 193 years, the NDVI ranged from 0.679 to 0.686, with a median of 0.683; the mean was 0.683, and the standard deviation (SD) was 0.002. 

There were 32 years with high vegetation greenness accounting for 16.6% of the whole series, while 37 years had low values, accounting for 19.2%. In order to demonstrate the NDVI variation at low frequencies, we performed 20-year low-pass filtering. At low frequencies, the NDVI changes were relatively stable. The vegetation greenness dynamics were relatively higher in the 1870s, 1930s, 1950s, and 1980s–1990s, while cover was lower in the 1850s–1860s, 1920s, 1960s–1970s, and 2000s–2010s (Table 4, Figure 6b).

The mean NDVI reconstruction was evaluated for both high and low vegetation greenness characteristics using the magnitude (the sum of deviations from the long-term median) and intensity (the sum of deviations from the median divided by the duration) of each event (three years or longer) over time [35]. Table 4 illustrates the magnitude and intensity of extreme vegetation greenness dynamics. In the NDVI reconstruction, the longest duration of high vegetation greenness occurred in 1980–1995 for a duration of 16 years; the longest duration of low vegetation greenness occurred in 2002–2012 for a duration of 11 years. In terms of the intensity of extreme events, the intensity of the nine events over 200 years did not vary significantly. Precipitation plays an extremely important role in the vegetation growth in the study area, and it can be seen in Figure 8 that the moisture in the study area continuously increases after 1980, which is undoubtedly extremely beneficial for vegetation growth. Similarly, after the 21st century, the PDSI values in the study area continued to decline, and the lack of available water for the plants caused a decrease in the NDVI values. In this regard, human activities also have an important influence, which is discussed in detail in Section 3.4.

**Figure 7:**
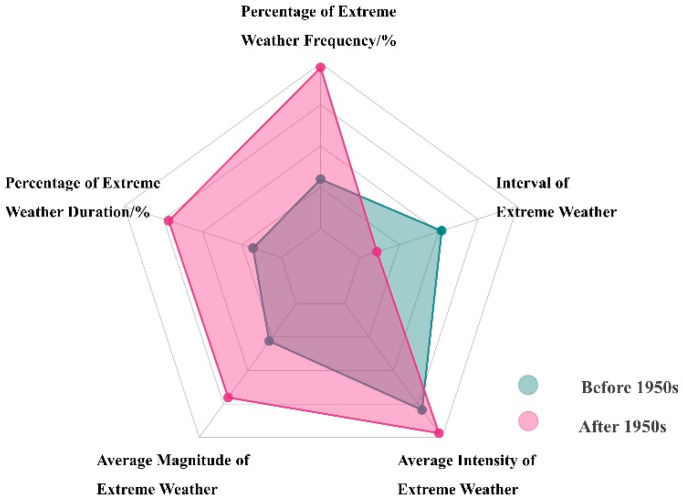
Comparison of the frequency, interval, intensity, scale, and duration of extreme events before and after the 1950s.

The sixth report of the IPCC states that the pattern of climate change since the mid-20th century may be caused by human activities, with adverse effects on vegetation greenness dynamics and agricultural production [1]. In response to this view, we analyzed the extreme changes in vegetation greenness dynamics around the 1950s, and found that the percentage of extreme events (number of extreme events/number of corresponding years) increased by 84.4%; the percentage of the duration of extreme events (the duration of extreme events/number of corresponding years) increased by 166.0%; the average size of extreme events increased by 68.9%; the average intensity increased by 6.2%, and the average extreme interval decreased by 57.2% (Figure 7). This indicates that after the 1950s, extreme changes in vegetation greenness dynamics have increased significantly in both magnitude and intensity, and this pattern is occurring more frequently.

Human-induced climate change has affected many weather extremes and climates in every region of the globe. Since the IPCC Sixth Assessment Report, the evidence for observed extreme weather changes such as heat waves, heavy precipitation, droughts, and tropical cyclones attributed to human activities has strengthened [1]. Evidence suggests that human activity has been a major driver of the frequency of extreme events since the 1950s [36]. Table 4 illustrates the magnitude and intensity of extreme vegetation greenness dynamics; this series of results may indicate that the intensity of extreme changes in vegetation greenness dynamics will increase if regional temperatures continue to rise.

### 3.3. The Impact of Climate Change on Vegetation Greenness Dynamics

**Figure 8:**
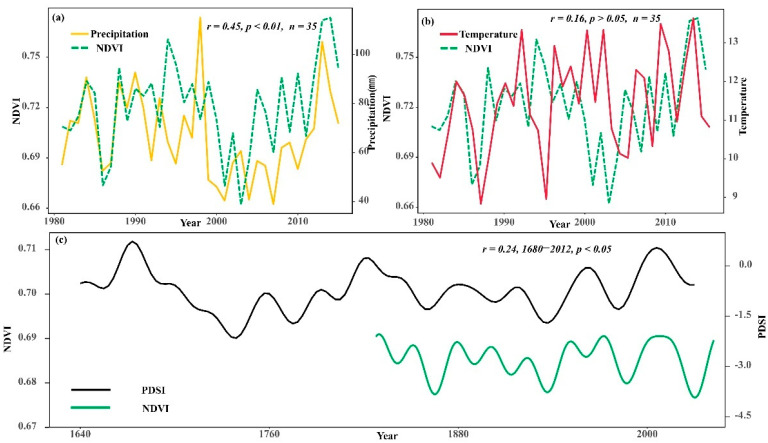
Relationship between the summer NDVI and precipitation, temperature, and the Palmer Drought Severity Index (PDSI). The NDVI and observed precipitation (**a**) and temperature (**b**) in the growing season; the NDVI and PDSI in Hulunbuir (**c**). (**c**) shows the correlation analysis for the common time period only, due to the different time lengths: 1826–2012.

The rise in vegetation greenness is influenced by regional temperature variation and hydrological conditions, and the NDVI in this study area was significantly correlated with precipitation (*r* = 0.45, *p* < 0.01) and not particularly strongly correlated with temperature (*r* = 0.16, *p* > 0.05) during 1952–2019 (Figure 8a,b). This result suggested that precipitation is the main factor limiting the change in vegetation greenness dynamics during the growing season.

Further study found that the summer NDVI in the Hulunbuir area was significantly correlated with the PDSI reconstruction [37] (*r* = 0.24, *p* < 0.05, Figure 8c). The PDSI can reflect both temperature and precipitation variability characteristics. The significant relationship between the two indicates that climate change is an important factor influencing the changes of summer vegetation greenness dynamics. Precipitation can affect the water content of the soil, which in turn affects the radial growth of trees [38]. Drought has a significant impact on the physiological activities of trees, directly affecting photosynthesis and indirectly affecting respiration and transpiration, causing plants to lack sufficient water, leading to partial dieback and reduced vegetation greenness [39,40].

### 3.4. The Impact of Human Activities on Vegetation Greenness Dynamics

Multiple regression residual analysis was used to study the effects of climate change and human activities on changes in the NDVI [41,42]. The formula is as follows:

NDVI_climate_ = 0.485136 + 0.001405 × P_P8C7_ + 0.006804 × T_C7_ + 0.000229 × P_P6_ (7)

where P_P8C7_ is the average precipitation from August of the previous year to July of the current year; T_C7_ is the average temperature in July of the current year, and P_P6_ refers to the precipitation in June of the previous year. The statistical parameters characterizing the NDVI_climate_ reconstruction equation are shown in Table 5.

**Figure 9:**
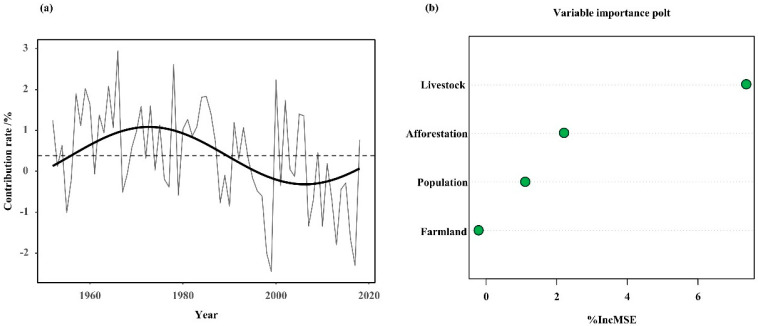
Contribution of human activities to dynamic changes in vegetation greenness dynamics (**a**). Ranking of the importance of factors influencing changes in vegetation greenness dynamics (**b**). The X-axis of Figure 9a is time, and the Y-axis is the contribution of human activities to the NDVI in the study area; the X-axis of Figure 9b is “%IncMSE”, which means the increase in the mean squared error, and the Y-axis is the importance of various types of human activities.

The study of the NDVI share influenced by human activities revealed that the positive human impact on vegetation gradually diminished after the founding of the People’s Republic of China (Figure 9a). After entering the 21st century, a fundamental reversal occurred, shifting from a positive to a negative impact. The contribution rate increased from 0.53% before 1949 to 0.80% (Figure 9a), and the impact of human activities increased year by year. For human activities, we screened some of the factors and conducted importance ranking analysis, and found that for vegetation greenness dynamics, the order was as follows: total livestock > afforestation area > population > farmland (Figure 9b).

The human-influenced vegetation greenness dynamics showed a sharp decline in 1987 and 1998, and only gradually recovered in later years. We learned by checking the data for fires from the Inner Mongolia Fire Brigade (https://www.119.gov.cn/xinwen/gddt/nmxf, accessed on 1 October 2021) that two huge forest fires, occurring in 1987 and 1998 in the Greater Khingan Range, led to the burning of large areas of forest, drastic damage to the local ecological environment, and a dramatic decrease in forest cover. After 2002, the impact of human activities on the vegetation greenness dynamics turned positive and the forest cover steadily increased, mainly due to the national policy of returning farmland to forest and grassland implemented in 2002, which gradually restored plots reclaimed as farmland or other use to forest and grassland, resulting in an increase in vegetation greenness after entering the 21st century [43]. Nevertheless, continued population growth and industrial development have, in turn, negatively impacted both forest and grassland vegetation habitats to varying degrees, encroaching on forest and grassland on a large scale, leading to a decline in vegetation greenness dynamics and leaving the entire region in a state of long-term fluctuation [44,45]. Notably, after 2012 the vegetation greenness dynamics displayed a strong upward trend that we attribute to the Chinese government’s insistence on prioritizing conservation (Figure 6 and Figure 9a), adhering to the basic state policy of conserving resources, protecting the environment, and promoting the concept of green development and lifestyle in the course of environmental protection.

The analysis of the importance of the influencing factors indicated that the total livestock and arable land area were the two most important human influences on vegetation greenness dynamics in the study area [46,47]. The implementation of the “grass–livestock dual contract” system in the Hulunbuir grassland pastoral area has led to the rapid development of the Hulunbuir agriculture and livestock industry, squeezing the area of forests and grasslands and causing ecological damage [48,49].

The influence of climatic and anthropogenic factors on the evolution of vegetation in the study area is bidirectional. Over the last two centuries, the contribution of the climate to the rise of the NDVI in the study area has shown a weak decreasing trend, while anthropogenic factors have shown a weak increasing trend, indicating that the influence of natural factors on the vegetation ecology of the study area is gradually decreasing but still dominant. The change of the vegetation greenness in the study area is the result of the combined effect of climate change and human activities. Vegetation greenness dynamics are dominated by climate change, and the decrease of the soil moisture in the study area caused by climate warming is the decisive factor.

## 4. Conclusions

Based on the chronology of *Pinus sylvestris* var. *mongolica* Litv. tree-ring widths, the NDVI of vegetation greenness dynamics was reconstructed for the growing season (May–September) in the western forest–grass transition zone area of the Greater Khingan Range in northeast China from 1826 to 2018, and the reconstructed equations passed stability and reliability tests. Among the past 193 years, year 32 showed higher vegetation greenness and year 37 showed lower vegetation greenness. Under low-pass filtering, the cover was relatively high in the 1870s, 1930s, 1950s, and 1980s–1990s, and low in the 1850s–1860s, 1920s, 1960s–1970s and 2000s–2010s. Climatic factors, especially precipitation, are the main factors influencing the changes in the vegetation greenness dynamics in summer. Climatic influences on vegetation greenness dynamics have occurred not only in recent decades but also in past centuries. The degree of influence of human activities on vegetation greenness dynamics has gradually increased since the 1950s, with the number of grazing livestock playing an important role in the changes in vegetation greenness dynamics, followed by the area of cultivated land, the total population, and the area of afforestation. Among the impacts of human activities, the order of importance was as follows: livestock > afforestation > population > farmland. Due to the influx of population after the founding of the People’s Republic of China, the grazing density and reclaimed area increased, destroying a large amount of vegetation and leading to a decrease in the NDVI values. Since the government-implemented project of returning farmland to forest and grass, the vegetation greenness dynamics in the study area have improved. These findings help us to understand the relationship between climate change and vegetation greenness dynamics in northeastern China, as well as the impact of human activities on vegetation greenness dynamics.

## Figures and Tables

**Table 1:** Basic information about the three locations where the tree cores were collected.

Sampling Sites	Haila’er	Guangtoushan	Huihe Forest
Sample code	HLE	GTS	HHF
Elevation (m) a.s.l.	655	770	700
Longitude (E)	119.690238	119.980839	119.663920
Latitude (N)	49.194272	48.242293	48.078830
Sample depth (core/tree)	72/46	33/16	54/30

**Table 2:** Basic information on the chronology of *Pinus sylvestris* var. *mongolica* Litv. tree rings at three locations.

Sampling Sites	Time Span (Year)	Mean Sensitivity (MS)	Series Intercorrelation	Expressed Population Signal (EPS)	Signal-to-Noise Ratio (SNR)	Standard Deviation (SD)	EPS > 0.85 in the First Year (Number of Trees)
HLE	1777–2019, 243	0.264	0.437	0.978	44.906	0.280	1817 (6)
GTS	1828–2019, 192	0.178	0.715	0.890	8.065	0.273	1898 (15)
HHF	1822–2019, 198	0.195	0.442	0.957	22.033	0.220	1886 (10)

Notes: Time span (Year) is the starting and ending times of the tree-ring chronology, and the number inside the brackets is the length of the tree-ring chronology (years). The first year where EPS > 0.85 (number of trees) is the year in which EPS first started to be greater than 0.85, and the number inside the parentheses is the number of tree cores with EPS > 0.85 years. HLE, Haila’er; GTS, Guangtoushan; HHF, Huihe Forestry.

**Table 3:** Statistics of the calibration and verification tests of the NDVI_total_ reconstruction.

Calibration				Verification				
Period	*r*	*R* ^2^	*F*	Period	RE	CE	ST	ST_1_
1982–1998	0.51	0.26	4.55	1999–2015	0.35	0.35	12+/5−	12+/4−
1999–2015	0.62	0.39	6.94	1982–1999	0.04	0.03	13+/4− *	11+/5−
1982–2015	0.55	0.31	6.81					

Note: “*” refers to α < 0.05.

**Table 4:** Reconstruction of low vegetation greenness and high vegetation greenness periods, and their scale and intensity in the western Greater Khingan Range.

Extreme Events	Period	Duration	Magnitude	Intensity	Extreme Events	Period	Duration	Magnitude	Intensity
Low NDVI values	1855–1864	10	−0.033	−0.003	High NDVI values	1872–1874	3	0.006	0.002
	1919–1928	10	−0.034	−0.003		1935–1939	5	0.012	0.002
	1966–1971	6	−0.016	−0.003		1952–1959	8	0.022	0.003
	2002–2012	11	−0.039	−0.004		1980–1995	16	0.048	0.003

**Table 5:** NDVI_climate_ reconstruction equation characteristic statistical parameters.

r	R^2^	$R_{\mathrm{adj}}^{2}$	p-Value
0.68	0.47	0.42	<0.01

Notes: $R_{\mathrm{adj}}^{2}$ refers to the amount of variance explained after adjusting for degrees of freedom. It removes the effect of the number of independent variables on *R*^2^, such that the magnitude of *R*^2^ only responds to the goodness of fit of the regression equation.

## Data Availability

The meteorological data can be downloaded at http://data.cma.cn/, accessed on 21 June 2021; NDVI data at http://data.tpdc.ac.cn/en/data/, accessed on 21 June 2021; and the fire data can be downloaded at https://www.119.gov.cn/xinwen/gddt/nmxf, accessed on 1 October 2021.
